# A case report of refractory angina in a patient with diabetes and apical hypertrophic cardiomyopathy

**DOI:** 10.1093/ehjcr/ytac347

**Published:** 2022-08-16

**Authors:** Nicholas Jex, Amrit Chowdhary, Sharmaine Thirunavukarasu, Eylem Levelt

**Affiliations:** Multidisciplinary Cardiovascular Research Centre and Biomedical Imaging Science Department, Leeds Institute of Cardiovascular and Metabolic Medicine, University of Leeds, Woodhouse, Leeds LS2 9JT, UK; Multidisciplinary Cardiovascular Research Centre and Biomedical Imaging Science Department, Leeds Institute of Cardiovascular and Metabolic Medicine, University of Leeds, Woodhouse, Leeds LS2 9JT, UK; Multidisciplinary Cardiovascular Research Centre and Biomedical Imaging Science Department, Leeds Institute of Cardiovascular and Metabolic Medicine, University of Leeds, Woodhouse, Leeds LS2 9JT, UK; Multidisciplinary Cardiovascular Research Centre and Biomedical Imaging Science Department, Leeds Institute of Cardiovascular and Metabolic Medicine, University of Leeds, Woodhouse, Leeds LS2 9JT, UK

**Keywords:** Cardiac magnetic resonance, Case report, Diabetes, Hypertrophic cardiomyopathy

## Abstract

**Background:**

Using serial imaging over time, this case reviews the natural history of co-morbid Type two diabetes (T2D) and apical hypertrophic cardiomyopathy (HCM) and assesses the potential combined impact on myocardial structure and perfusion.

**Case summary:**

A 59-year-old patient with concomitant T2D and an apical phenotype of HCM was seen over a 11-year period with a significant burden of anginal chest pain. Chest pain was refractory to anti-anginal medical therapy and persisted at on-going follow-up. Multi-modality imaging demonstrated significant deterioration in coronary microvascular function and increased myocardial scar burden despite unobstructed epicardial coronary arteries.

**Discussion:**

Comorbidity with T2D and apical HCM resulted in a significant increase in myocardial fibrosis and deterioration in coronary microvascular function.

**Learning points**

- To highlight the role of coronary microvascular dysfunction in patients with co-morbid Type 2 diabetes (T2D) and apical hypertrophic cardiomyopathy (HCM) presenting with chest pain
- To review the natural history of co-morbid T2D and apical HCM with serial cardiac magnetic resonance imaging
- To highlight the potential for significant interval increases in myocardial scar burden in apical HCM despite ESC 5-year sudden cardiac death risk remaining low

## Introduction

Cardiac magnetic resonance (CMR) allows for a non-invasive assessment of coronary microvascular function and has been validated against coronary angiography and invasive physiology.^1^

Quantification of global and regional stress and rest myocardial blood flow (mL/g/min) can be calculated with CMR using knowledge of the amount of contrast present in tissue and the arterial input function driving delivery of the contrast agent.^2^ The myocardial perfusion reserve (MPR) is the ratio of stress to rest MBF, both MBF and MPR have been shown to be strong independent predictors of cardiovascular outcomes and mortality.^1^

We present a case of chronic refractory chest pain with deteriorating coronary microvascular function and increasing myocardial scar burden despite the absence of significant epicardial coronary artery disease on repeated investigations.

## Timeline

**Table:** 

Time	Events
26 April 2010	Admitted to hospital with acute chest pain in context of non-elevated troponin. ECG shows antero-lateral T wave inversion
28 April 2010	Diagnostic coronary angiography shows non-obstructed epicardial coronary arteries
21 June 2010	Baseline cardiac MRI confirms apical HCM phenotype
2 July 2014	Re-admission with acute chest pain with elevated Troponin
3 July 2014	Repeat coronary angiography shows non-obstructed epicardial coronary arteries
15 May 2015	Diagnosed Type 2 diabetes
26 June 2015	Seen in cardiology clinic with stable angina. Commenced on beta-blocker
29 September 2016	Addition of Verapamil due to refractory angina
13 July 2017	Repeat cardiac MRI study due to refractory symptoms. Worsening microvascular function and progression of scarring
14 April 2018	Addition of isosorbide mononitrate due to refractory angina
15 August 2018	Admitted to hospital with chest pain and elevated troponin
27 December 2018	Addition of ranolazine due to refractory angina
14 October 2019	Repeat cardiac MRI study shows worsening microvascular function and further progression of scar
26 June 2021	Further admission with chest pain and troponin elevation
27 June 2021	CT coronary angiogram shows non-obstructed coronary arteries
27 June 2021	Episode of non-sustained ventricular tachycardia on telemetry monitoring
30 September 2021	Seen in clinic; persisting angina. Commenced SGLT-I due to sub-optimal Type two diabetes control.
1 June 2022	On-going limiting anginal chest pain. Current medical therapy; Verapamil 120 mg, Isosorbide mononitrate 30 mg, Bumetanide 1 mg, Atorvastatin 20 mg, Aspirin 75 mg, Spironolactone 20 mg. On-going annual cardiology follow-up.

## Case presentation

### Patient information

A 59-year-old patient with concomitant Type 2 diabetes (T2D) and an apical phenotype of hypertrophic cardiomyopathy (HCM) was seen over an 11-year period at the Inherited Cardiac Conditions (ICCs) clinic. The patient was first diagnosed with HCM at the age of 49 (2010) following an admission to hospital with a troponin negative chest pain.

The patient was of South Asian heritage and there was no family history of HCM, other inherited cardiomyopathies, or sudden cardiac death (SCD). Co-morbidities included essential hypertension, previous lacunar stroke and obstructive sleep apnoea. The patient was subsequently diagnosed with T2D (2015). He was a lifelong non-smoker.

### Physical examination and diagnostic assessment

**Figure 1:**
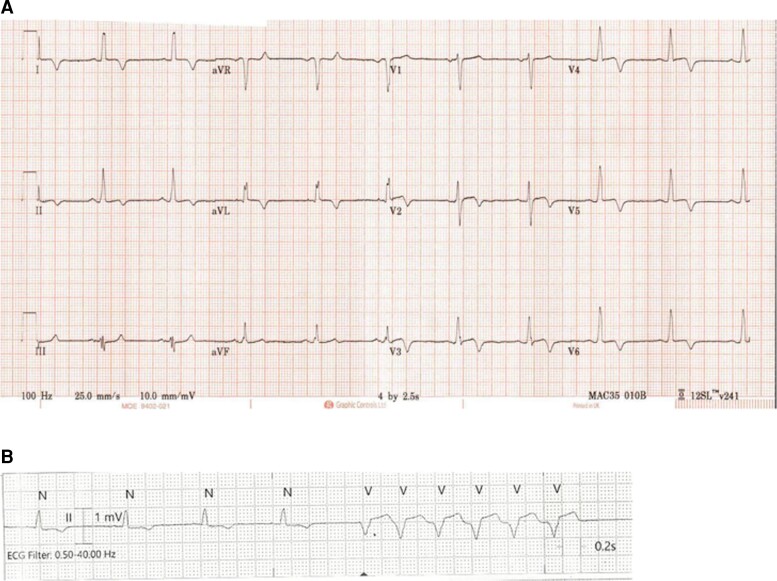
(*A*) Twelve-lead electrocardiogram showing antero-lateral T wave inversion characteristic of apical HCM and (*B*) episode of non-sustained VT.

A 12-lead electrocardiogram (ECG) showed sinus rhythm with antero-lateral T wave inversion characteristic of an apical HCM phenotype (*Figure 1A*). He had an elevated body mass index; clinical examination was otherwise unremarkable.

Given the presence of atherosclerotic risk factors and antero-lateral ECG changes the initial primary differential diagnosis was unstable angina.

Baseline Invasive coronary angiography in 2010 showed non-obstructed coronary arteries and echocardiography showed apical left ventricular (LV) hypertrophy with no LV outflow tract obstruction at rest or during hemodynamic stress with dobutamine stress echocardiography. He had an unremarkable 24-hour ECG monitor with no evidence of ventricular arrythmia.

The standard gene panel screening for 21 HCM genes did not reveal any causative sarcomeric mutation and European Society of Cardiology (ESC) 5-year SCD risk score was calculated at 1.76%.

Given the lack of a causative sarcomeric mutation on genetic testing, and in line with ESC guidelines,^3^ ECG and echocardiographic screening was undertaken in the patient’s one surviving first degree relative. However, this did not reveal any features of HCM.

Changes in clinical characteristics and CMR imaging findings over time are provided in *Table 1*.

**Table 1:** Changes in clinical and CMR parameters over time

Variable	2010	2017	2019
BMI, kg/m^2^	34	36	38
Heart rate, bpm	72	64	70
Systolic BP, mmHg	129	130	128
Diastolic BP, mmHg	79	74	84
eGFR, mL/min/1.73m^2^	70	64	61
HbA1c, mmol/mol	−	50	58
Medications			
Ramipril	−	−	
Bisoprolol	+	+	+
Diltiazem	+	+	+
Spironolactone	+	+	+
Aspirin	+	+	+
DOAC	−	−	
Metformin	−	+	+
Atorvastatin	−		+
DPP4i	−		−
GLP-1RA	−		−
Empagliflozin	−		+
Ranolazine	−	−	+
Clinical features			
NSVT	−	−	+
NYHA Class	III	III	III
ESC risk score (%)	1.76	1.67	3.35
Angina Class	II	III	III
CMR parameters			
LV end-diastolic volume indexed to BSA, mL/m^2^	69	68	69
LV end-systolic volume indexed to BSA, mL/m^2^	17	18	24
LV mass, g	243	250	252
LV mass index, g/m^2^	103	106	107
LV mass to LV end-diastolic volume, g/mL	1.5	1.6	1.6
LV stroke volume, mL	120	113	96
LV ejection fraction, %	74	72	63
LV maximal wall thickness, mm	21	23	24
RV end-diastolic volume indexed to BSA, mL/m^2^	71	61	51
RV end-systolic volume indexed to BSA, mL/m^2^	28	23	21
RV stroke volume, mL	102	86	70
RV ejection fraction, %	61	60	58
Global longitudinal strain, negative (−), %	15	12	8
Peak circumferential diastolic strain rate, s^–1^	0.88	−	0.61
Mean native T1, (ms)	−	1351	1395
Extra cellular volume, (%)	−	31	38
LGE scar percentage of LV mass (%)	6	26	40
Stress MBF, mL/min/g	2.63	1.1	0.75
Rest MBF, mL/min/g	0.9	0.5	0.6
MPR	2.9	2.2	1.3

BMI, body mass index; bpm, beats per minute; BP, blood pressure; BSA, body surface area; T2D, Type 2 diabetes; HCM, hypertrophic cardiomyopathy; HR, heart rate; ESC, European Society of Cardiology; HbA1c, glycated haemoglobin; NSVT, non-sustained ventricular tachycardia; NYHA, New York Heart Association; LV, left ventricular; LA, left atrial; LA EF, left atrial ejection fraction; LGE, late gadolinium enhancement; MBF, myocardial blood flow; MPR, myocardial perfusion reserve; eGFR, estimated glomerular filtration rate; ACEI, angiotensin converting enzyme inhibitor; ARB, angiotensin receptor blocker; CCB, calcium channel blocker; ASA, aspirin; DOAC, direct oral anticoagulant; DPP4i, dipeptidyl peptidase-4 inhibitor; GLP-1RA, glucagon-like peptide-1 receptor agonist; MRA, mineralocorticoid receptor antagonist; SGLT2i, sodium–glucose co-transporter 2 inhibitor.

A baseline CMR scan was performed in 2010 with cine, adenosine stress perfusion and late gadolinium enhancement (LGE) imaging acquisitions to assess left ventricular (LV) mass, function, perfusion, and scar burden. Findings were concordant with the echocardiogram showing typical features of an apical HCM phenotype with maximal end-diastolic wall thickness of 21 mm measured at the apical septum and end-systolic mid-cavity obliteration at rest. The baseline perfusion images showed a marked global circumferential perfusion defect suggestive of coronary microvascular dysfunction.

### Intervention

Over the following 11 years the patient suffered with a significant burden of anginal symptoms which were refractory to standard anti-anginal therapy including calcium channel blockers, beta blockers, nitrates and ranolazine.

### Follow-up and outcomes

The patient required a total of 4 hospital admissions to acute cardiology services due to episodes of refractory chest pain with elevated but non-dynamic troponin changes. His diabetes control was persistently sub-optimal with a mean HbA1c of 54 mmol/mol.

**Figure 2:**
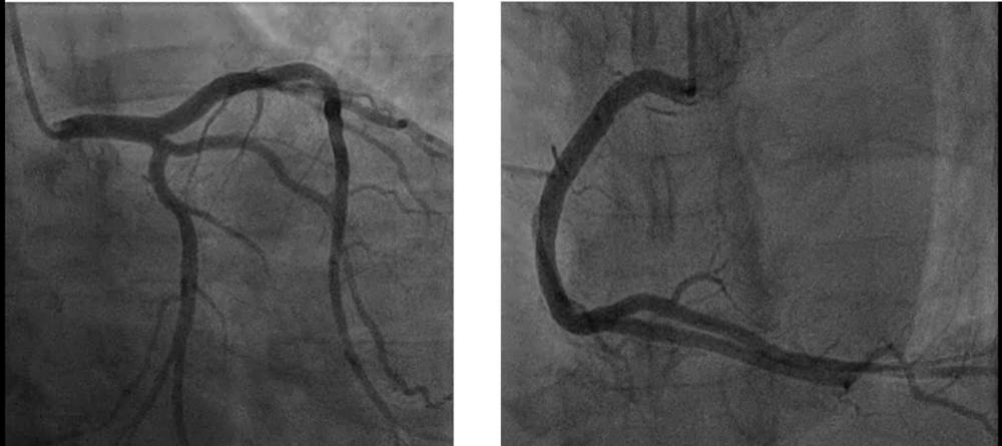
Representative images from invasive X-ray diagnostic coronary angiography showing unobstructed coronary arteries from 2014.

Despite the presence of multiple atherosclerotic risk factors, repeated invasive X-ray coronary angiography assessments in 2010 and 2014 and computed tomography coronary angiography in 2021 showed no significant obstructive atheroma (*Figure 2*).

**Figure 3:**
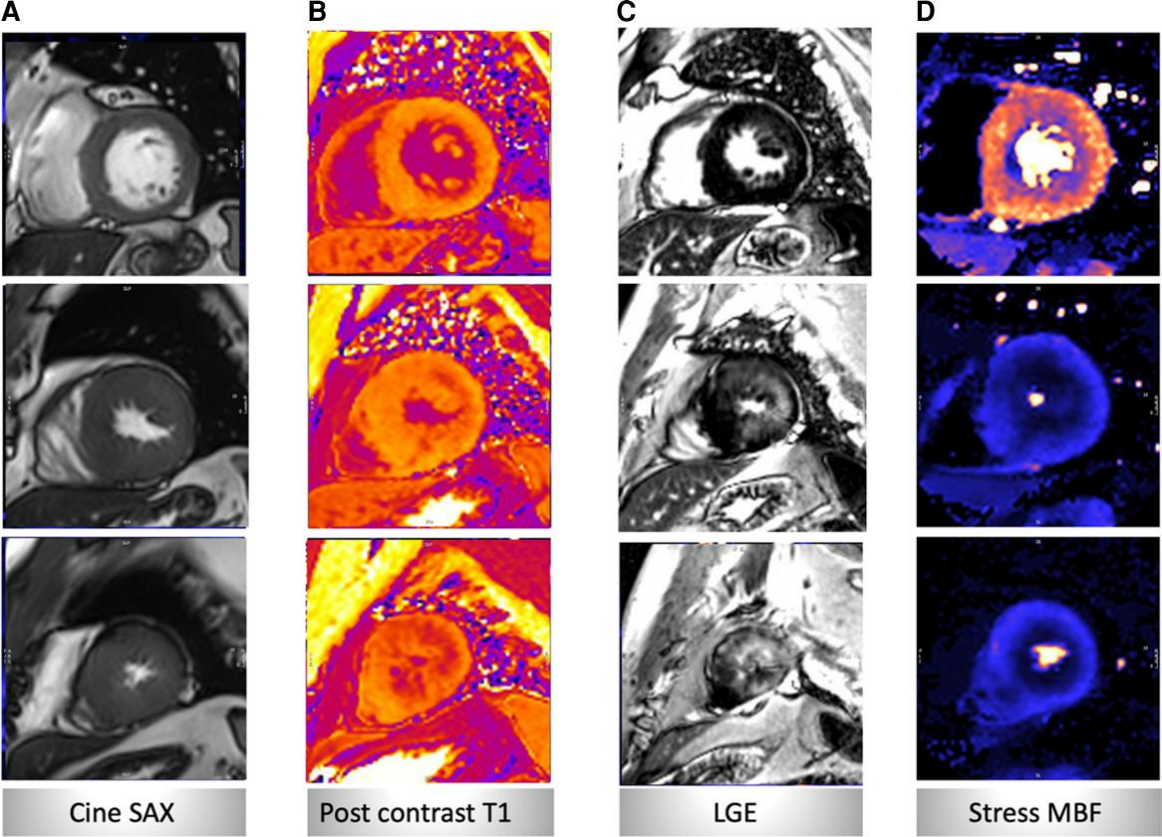
Example showing short axis basal, mid-ventricular and apical cine images (*A*), post-contrast T_1_ maps (*B*), late gadolinium enhancement (*C*) and stress myocardial blood flow (*D*).

**Figure 4:**
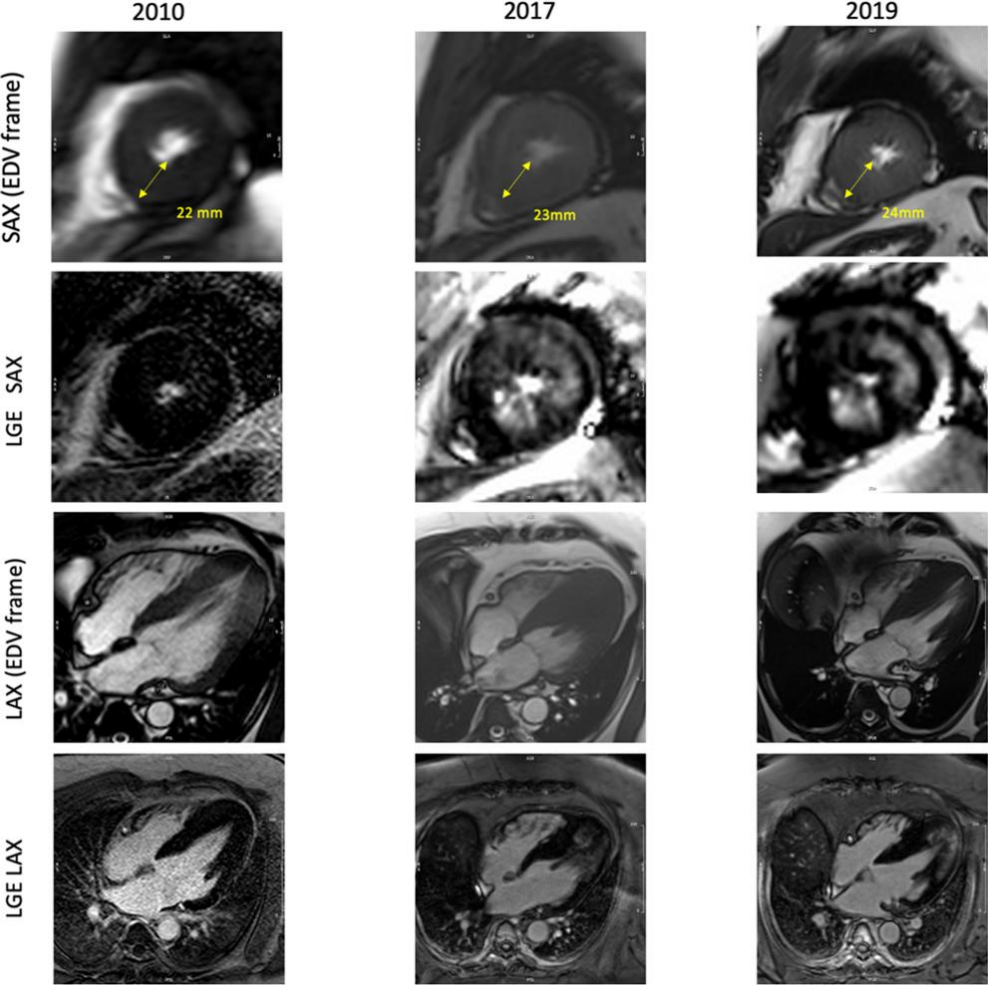
Changes in left ventricular late gadolinium enhancement (LGE) over time, images in the short axis (SAX) end-diastolic volume (EDV) frame and long axis (LAX) end-diastolic volume frame.

CMR imaging was repeated in 2017 and 2019 for further assessments due to the patient’s worsening symptomatic status. Quantitative perfusion analysis confirmed interim deterioration of the coronary microvascular function with a significant reduction in global stress myocardial blood flow (MBF) and in myocardial perfusion reserve (MPR) over time (*Figure 3*). While there were only small areas of patchy mid-wall hyperenhancement on the LGE at baseline CMR, the surveillance scans over 11 years showed a striking stepwise increase in the myocardial scar burden on LGE with scar percentage increasing from 6 to 40% on the final scan (*Figure 4*). LGE images showed no subendocardial hyperenhancement suggestive of a myocardial infarction. There was a stepwise interval decline in cardiac contractile function in left ventricular ejection fraction (LVEF) and global longitudinal strain (GLS) over time. There was a modest interval increase in maximal LV apical wall thickness and myocardial mass.

During the patient’s most recent admission in June 2021 telemetry showed an asymptomatic episode of non-sustained ventricular tachycardia (*Figure 1B*), with the ESC 5-year risk score for SCD increased to 3.35%.

Given the documented episode of NSVT and high myocardial scar burden implantation of ICD was considered by the ICC multidisciplinary team and discussed with the patient. Considering that the ESC 5-year SCD risk score remained below 5% and given the patient’s preference for a delay to device therapy until of clear benefit, it was felt that there was not yet a clear mandate for ICD implantation.

The patient’s chest pain remained refractory to medical therapy at most recent follow-up, he was commenced on sodium–glucose co-transporter protein-2 (SGLT-2) inhibitor due to his sub-optimal T2D control.

## Discussion

Diabetes occurs concomitantly in 9% of patients with HCM and is associated with worsened clinical manifestation of HCM.^4,5^ The prevalence of T2D comorbidity is higher in patients with an apical HCM phenotype compared to non-apical HCM phenotypes (15% vs. 7%, respectively), the reasons for this are not well understood.^4^ Although distinct entities, both T2D and HCM have been independently shown to be associated with coronary microvascular dysfunction.^6,7^

This case offers unique insight into the natural history of these co-morbid conditions with serial CMR imaging and perfusion quantification over the follow-up period. In registry studies HCM patients with T2D comorbidity were shown to have higher prevalence of diastolic dysfunction and pulmonary hypertension, higher New York Heart Association Class, lower exercise capacity and increased long-term mortality.^4^ The mechanisms for the adverse prognostic association between HCM and T2D are incompletely understood but may include the collective impact of HCM and T2D on myocardial perfusion and the fibrotic process. Our finding of deterioration in coronary microvascular function with a high symptom burden and a dramatic increase in myocardial scar burden in this case may support this theory. Recent HCM registry data showed LGE in apical HCM in 45.8% of subjects while the extent/presence of (apical or any) LGE does not feature in the ESC HCM risk-stratification algorithm.^3,8^ The significant increase in myocardial scar burden from 6 to 40% over 9 years with subsequent non-sustained ventricular arrythmia despite ESC 5-year SCD remaining below 5% also highlights the potential pitfalls of this risk stratification tool.

This case of co-morbid T2D and apical HCM highlights the potential for a deleterious effect on coronary microvascular function when these two conditions occur simultaneously. The resulting protracted symptoms of chest pain, which were refractory to current anti-anginal therapy, led to significant morbidity and repeated use of acute cardiology services. This suggests further evidence-based treatments for this sub-set of patients may be required.

The marked increase in myocardial scar burden and presence of non-sustained ventricular tachycardia despite ESC 5-year SCD remaining below 5% also highlights the potential pitfalls of this risk stratification tool in such cases.

## Supplementary Material

ytac347_Supplementary_Data

## Lead author biography

Nicholas Jex is a PhD student in cardiac MRI and cardiac metabolism at the university of Leeds. His research is in the use of magnetic resonance spectroscopy and CMR imaging to further understanding of the impact of diabetes in states of cardiac hypertrophy.

## Notes

**Slide sets:** A fully edited slide set detailing this case and suitable for local presentation is available online as Supplementary data.

**Consent:** The authors confirm that written consent for submission and publication of this case report including images and associated text has been obtained from the patient in-line with COPE guidelines.

**Funding:** None declared.
