# Supplementary material for: A case report of refractory angina in a patient with diabetes and apical hypertrophic cardiomyopathy
Source: Eur Heart J Case Rep. 2022 Aug 16;6(8):ytac347. doi: 10.1093/ehjcr/ytac347 (PMC9425848; doi:10.1093/ehjcr/ytac347)
Supplement: ytac347_Supplementary_Data [file ytac347_supplementary_data.zip › EHJ-CR-Slide-Set_final (1).pptx]

## Slide 1
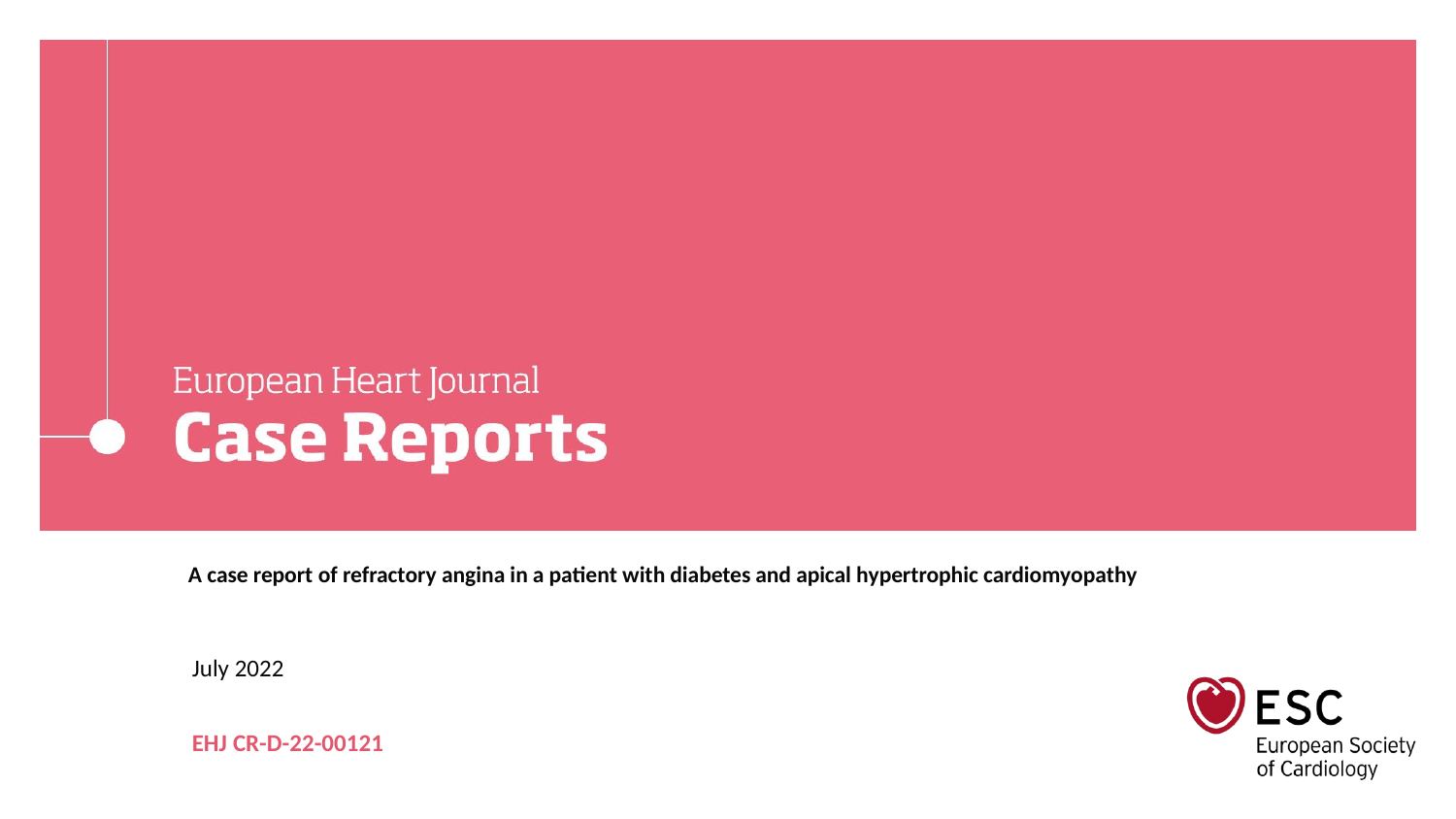

# A case report of refractory angina in a patient with diabetes and apical hypertrophic cardiomyopathy
July 2022
EHJ CR-D-22-00121

## Slide 2
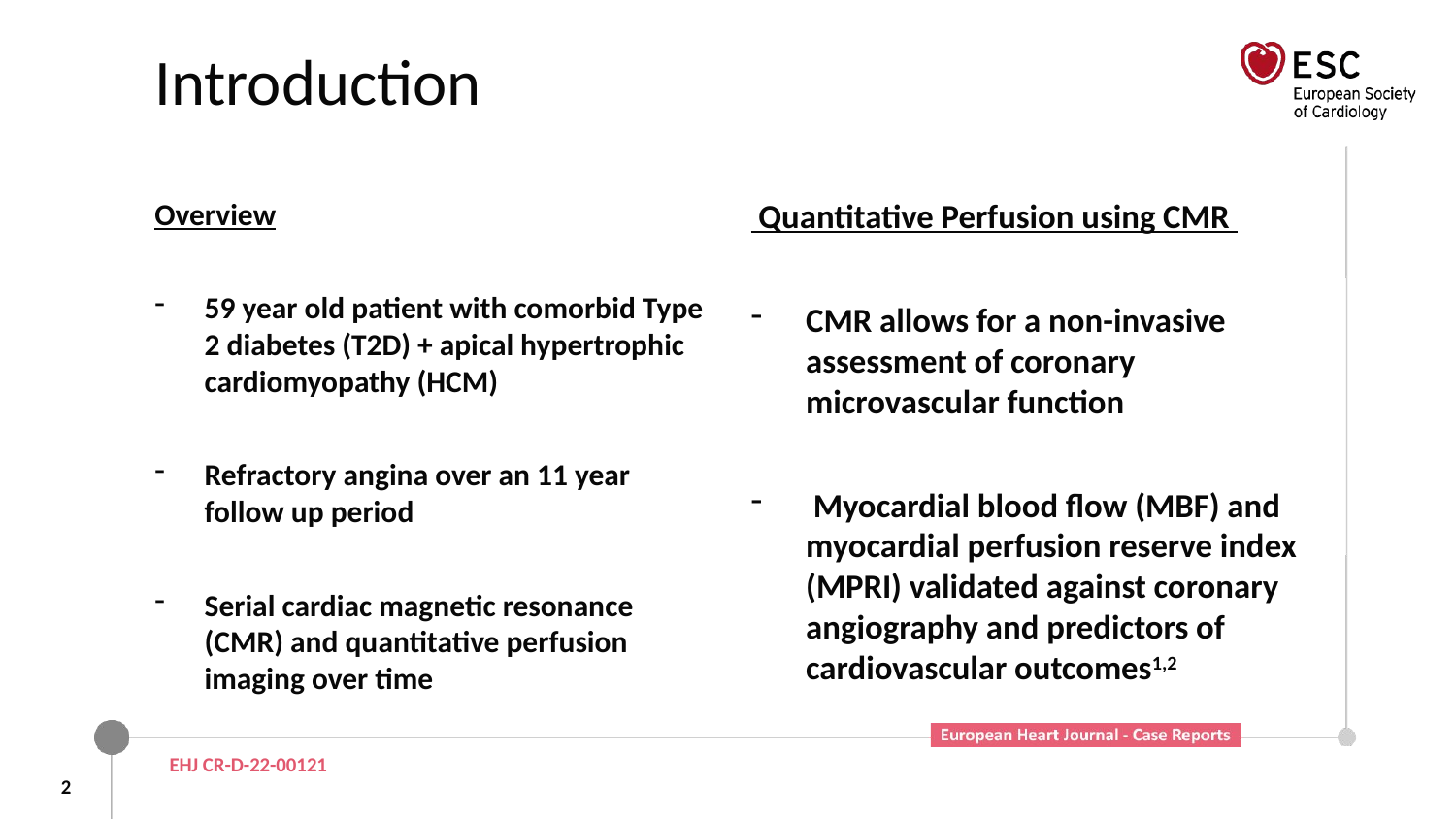

# Introduction
Overview
59 year old patient with comorbid Type 2 diabetes (T2D) + apical hypertrophic cardiomyopathy (HCM)
Refractory angina over an 11 year follow up period
Serial cardiac magnetic resonance (CMR) and quantitative perfusion imaging over time
 Quantitative Perfusion using CMR
CMR allows for a non-invasive assessment of coronary microvascular function
 Myocardial blood flow (MBF) and myocardial perfusion reserve index (MPRI) validated against coronary angiography and predictors of cardiovascular outcomes1,2
EHJ CR-D-22-00121
2

## Slide 3
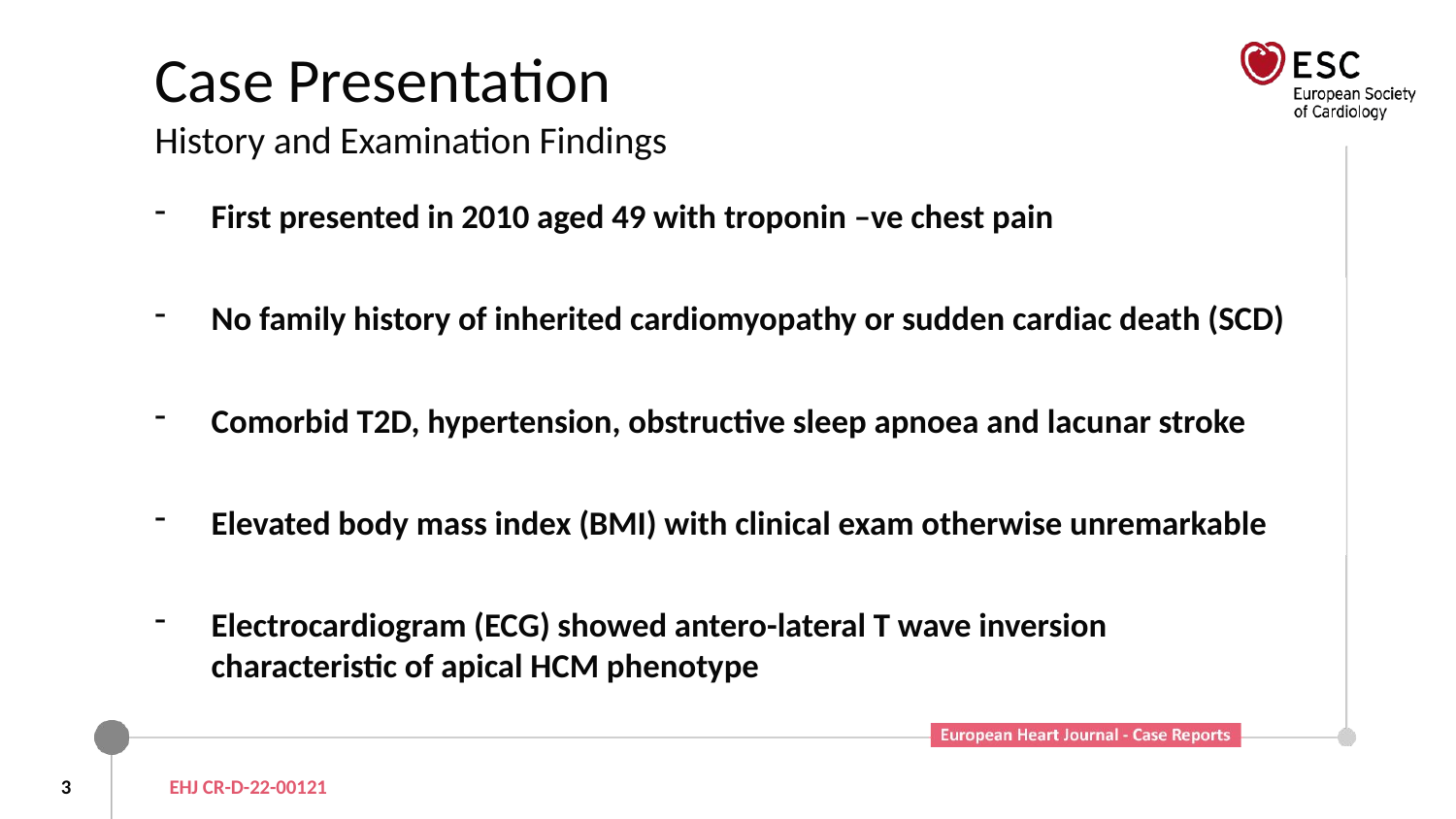

# Case Presentation History and Examination Findings
First presented in 2010 aged 49 with troponin –ve chest pain
No family history of inherited cardiomyopathy or sudden cardiac death (SCD)
Comorbid T2D, hypertension, obstructive sleep apnoea and lacunar stroke
Elevated body mass index (BMI) with clinical exam otherwise unremarkable
Electrocardiogram (ECG) showed antero-lateral T wave inversion characteristic of apical HCM phenotype
3
EHJ CR-D-22-00121

## Slide 4
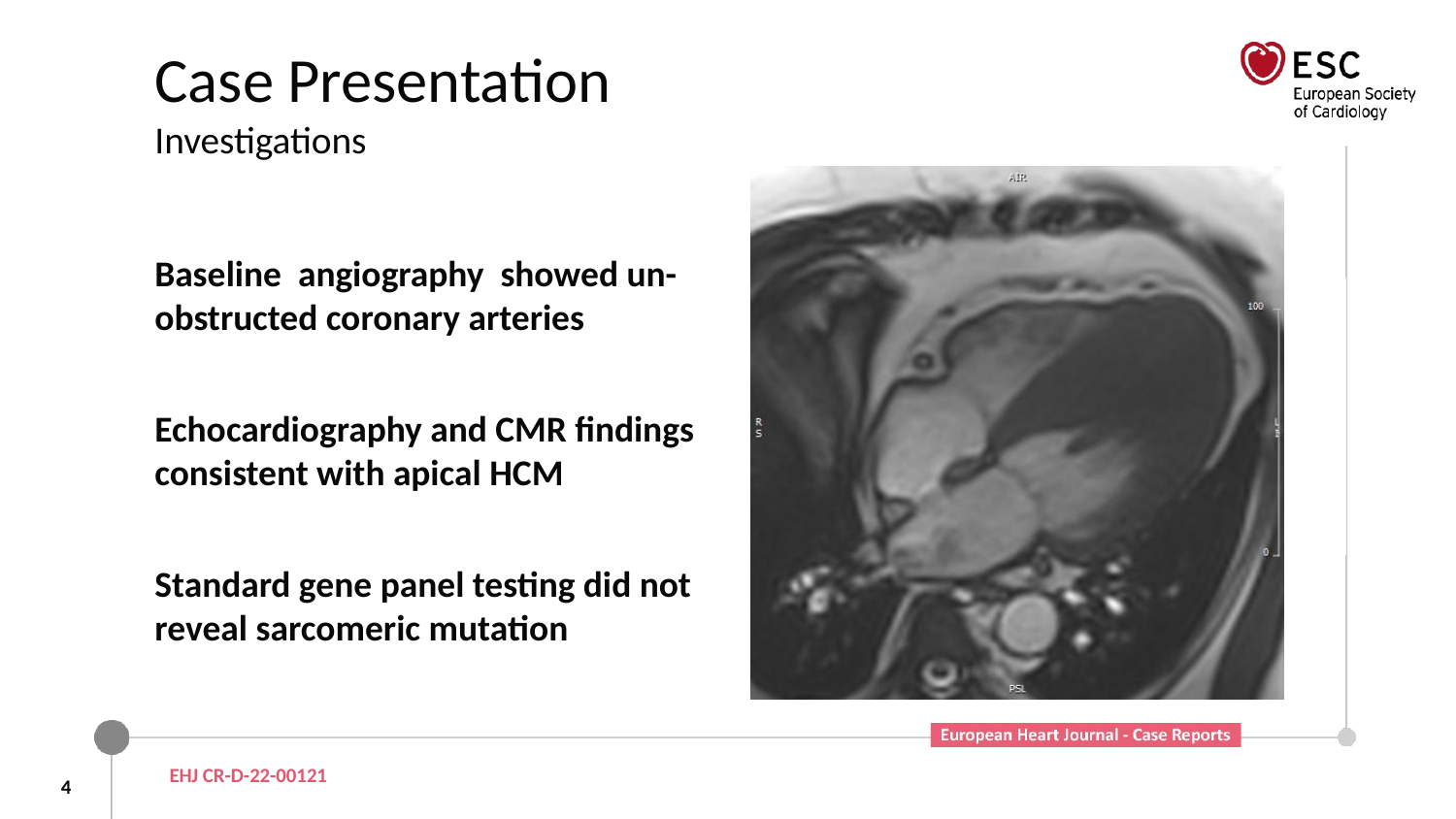

# Case Presentation Investigations
Baseline angiography showed un-obstructed coronary arteries
Echocardiography and CMR findings consistent with apical HCM
Standard gene panel testing did not reveal sarcomeric mutation
4
EHJ CR-D-22-00121

## Slide 5
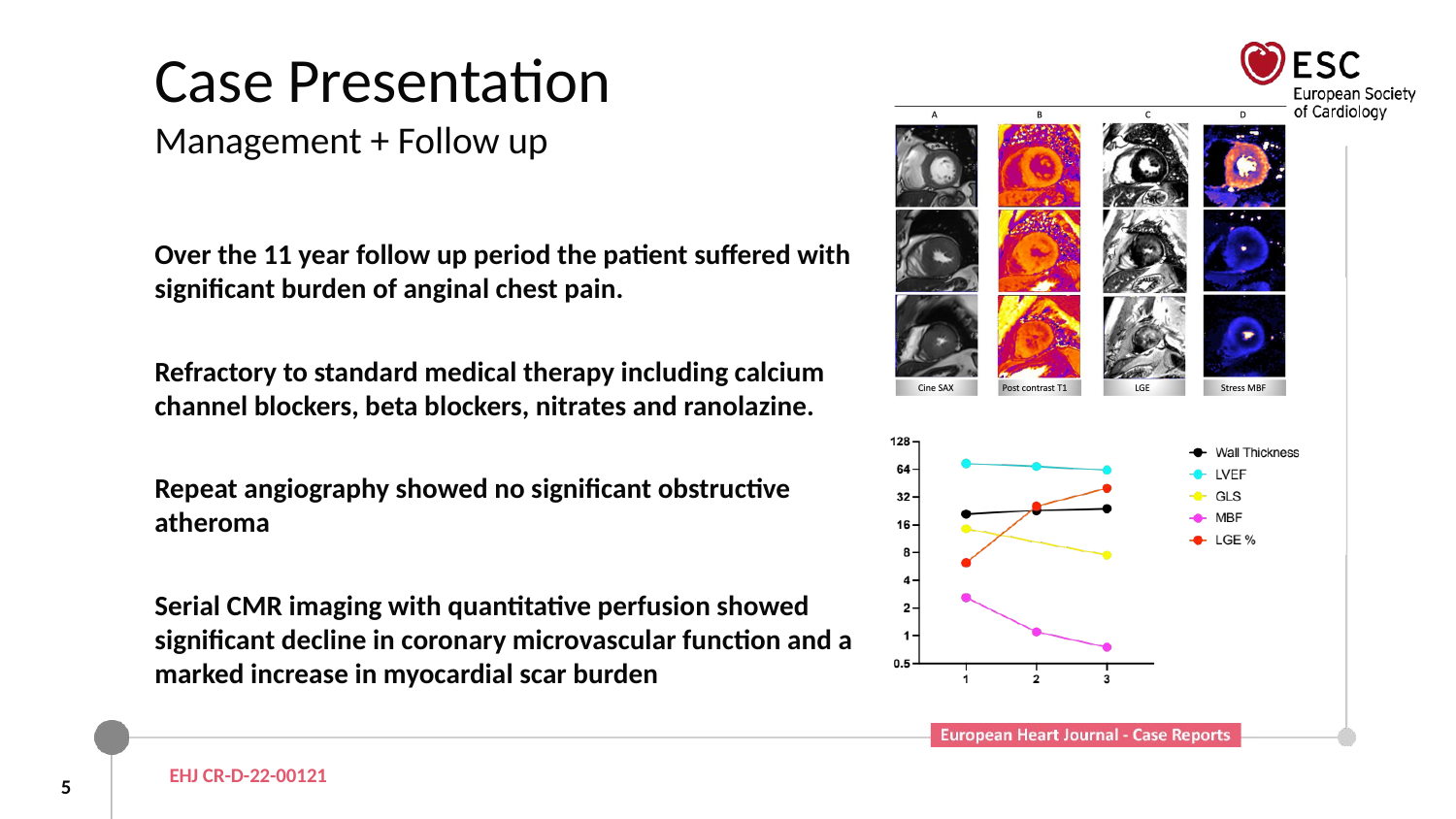

# Case Presentation Management + Follow up
Over the 11 year follow up period the patient suffered with significant burden of anginal chest pain.
Refractory to standard medical therapy including calcium channel blockers, beta blockers, nitrates and ranolazine.
Repeat angiography showed no significant obstructive atheroma
Serial CMR imaging with quantitative perfusion showed significant decline in coronary microvascular function and a marked increase in myocardial scar burden
5
EHJ CR-D-22-00121

## Slide 6
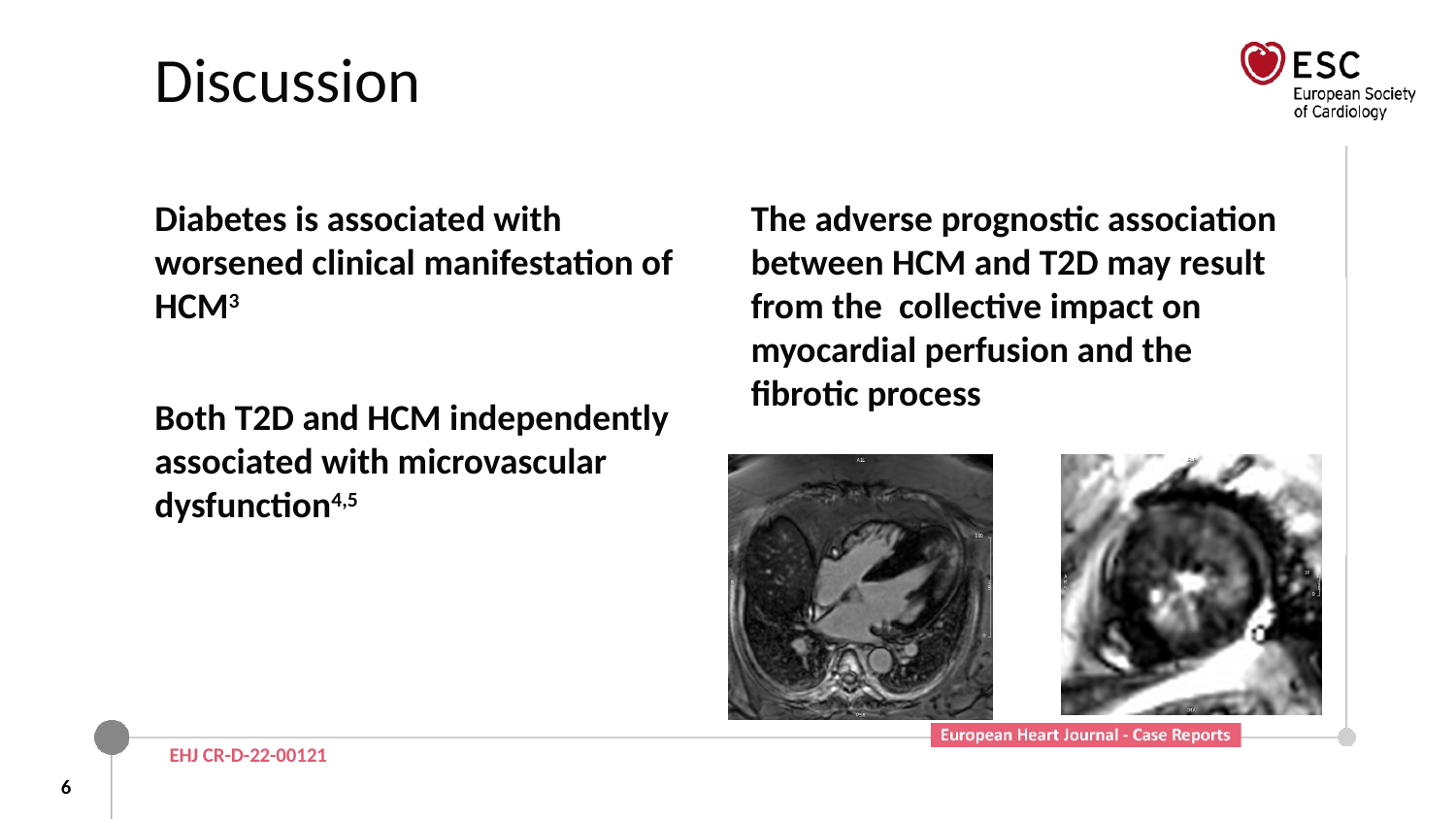

# Discussion
Diabetes is associated with worsened clinical manifestation of HCM3
Both T2D and HCM independently associated with microvascular dysfunction4,5
The adverse prognostic association between HCM and T2D may result from the collective impact on myocardial perfusion and the fibrotic process
EHJ CR-D-22-00121
6

## Slide 7
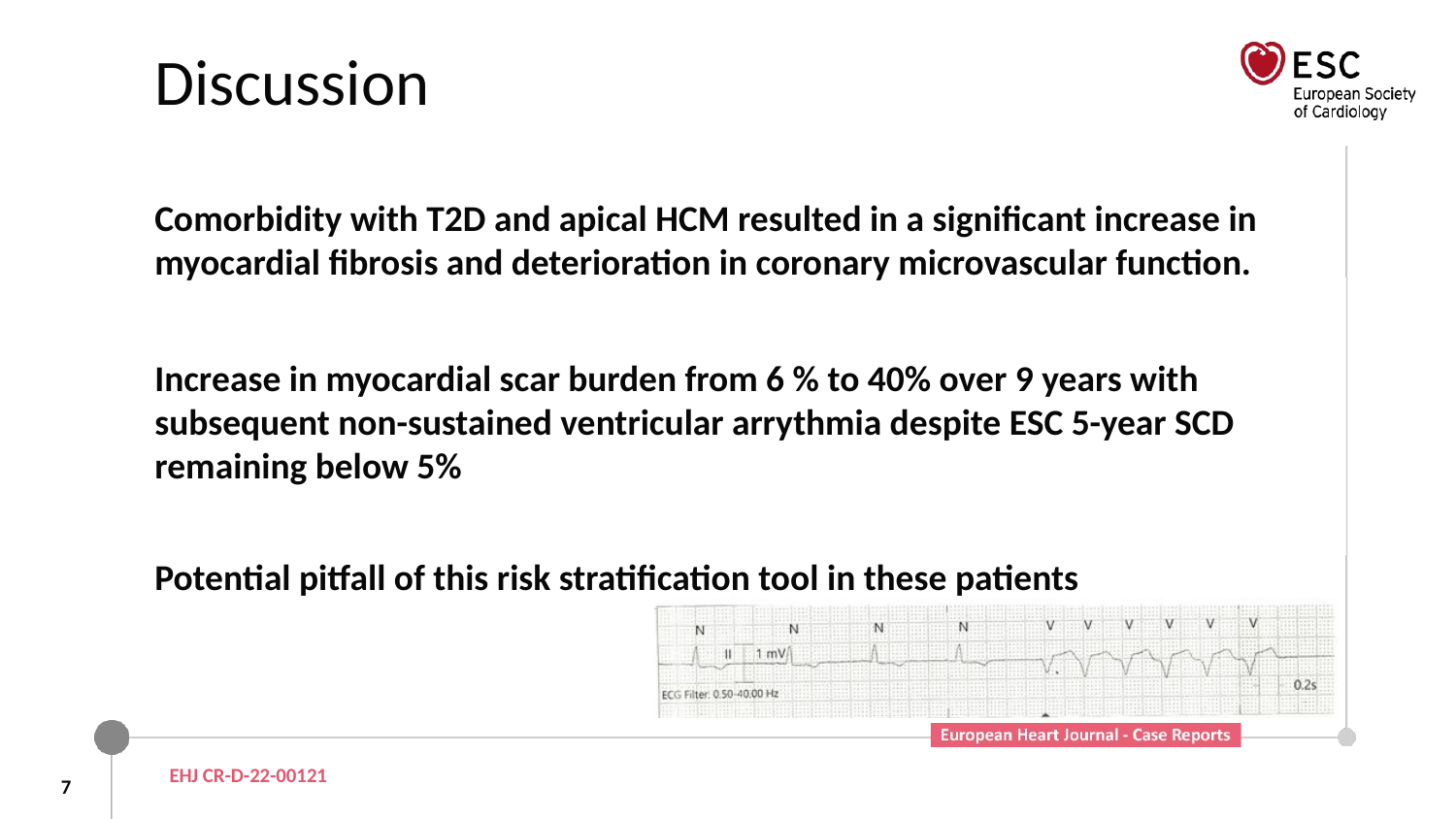

# Discussion
Comorbidity with T2D and apical HCM resulted in a significant increase in myocardial fibrosis and deterioration in coronary microvascular function.
Increase in myocardial scar burden from 6 % to 40% over 9 years with subsequent non-sustained ventricular arrythmia despite ESC 5-year SCD remaining below 5%
Potential pitfall of this risk stratification tool in these patients
7
EHJ CR-D-22-00121

## Slide 8
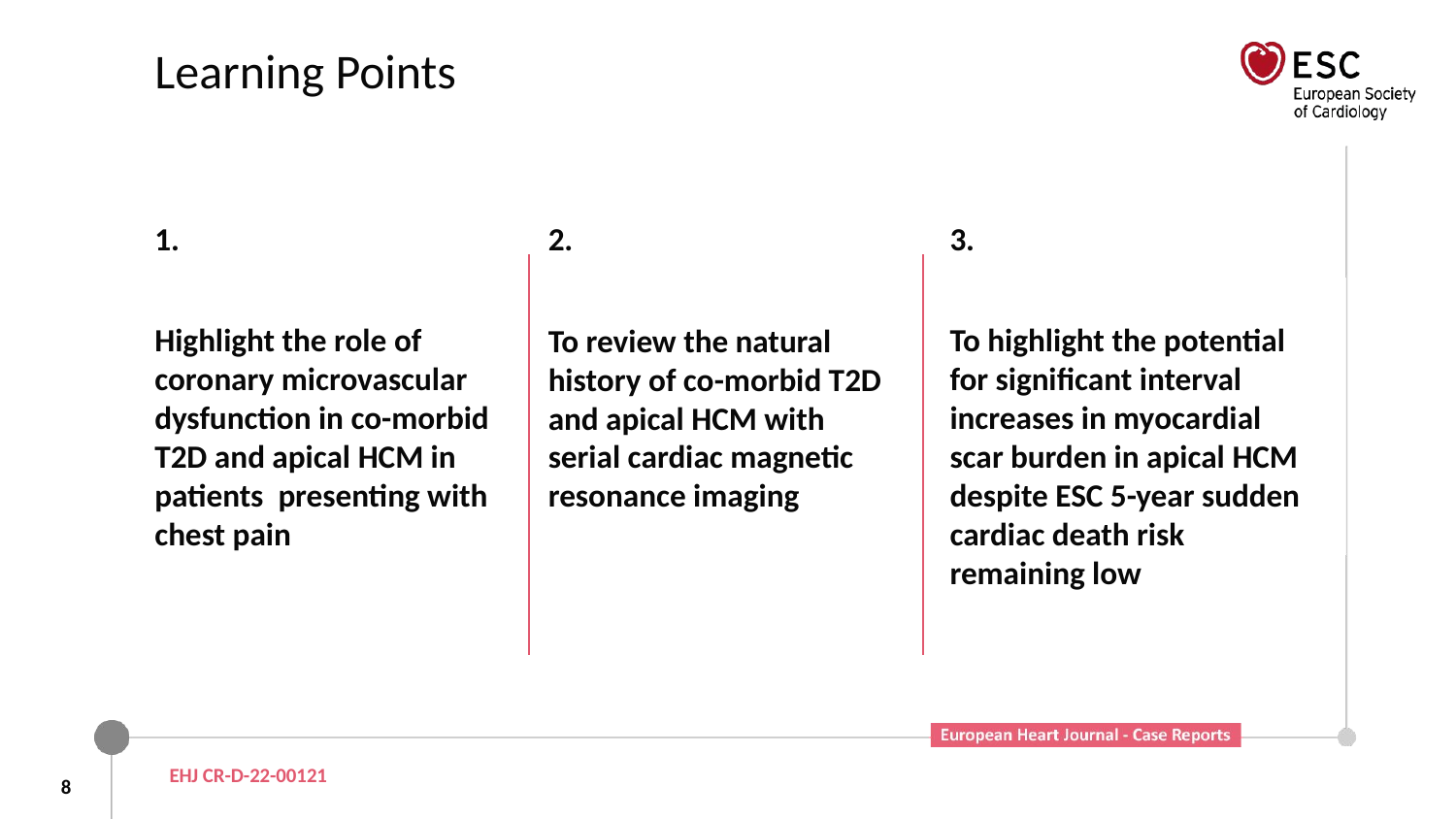

# Learning Points
1.
Highlight the role of coronary microvascular dysfunction in co-morbid T2D and apical HCM in patients presenting with chest pain
2.
To review the natural history of co-morbid T2D and apical HCM with serial cardiac magnetic resonance imaging
3.
To highlight the potential for significant interval increases in myocardial scar burden in apical HCM despite ESC 5-year sudden cardiac death risk remaining low
8
EHJ CR-D-22-00121

## Slide 9
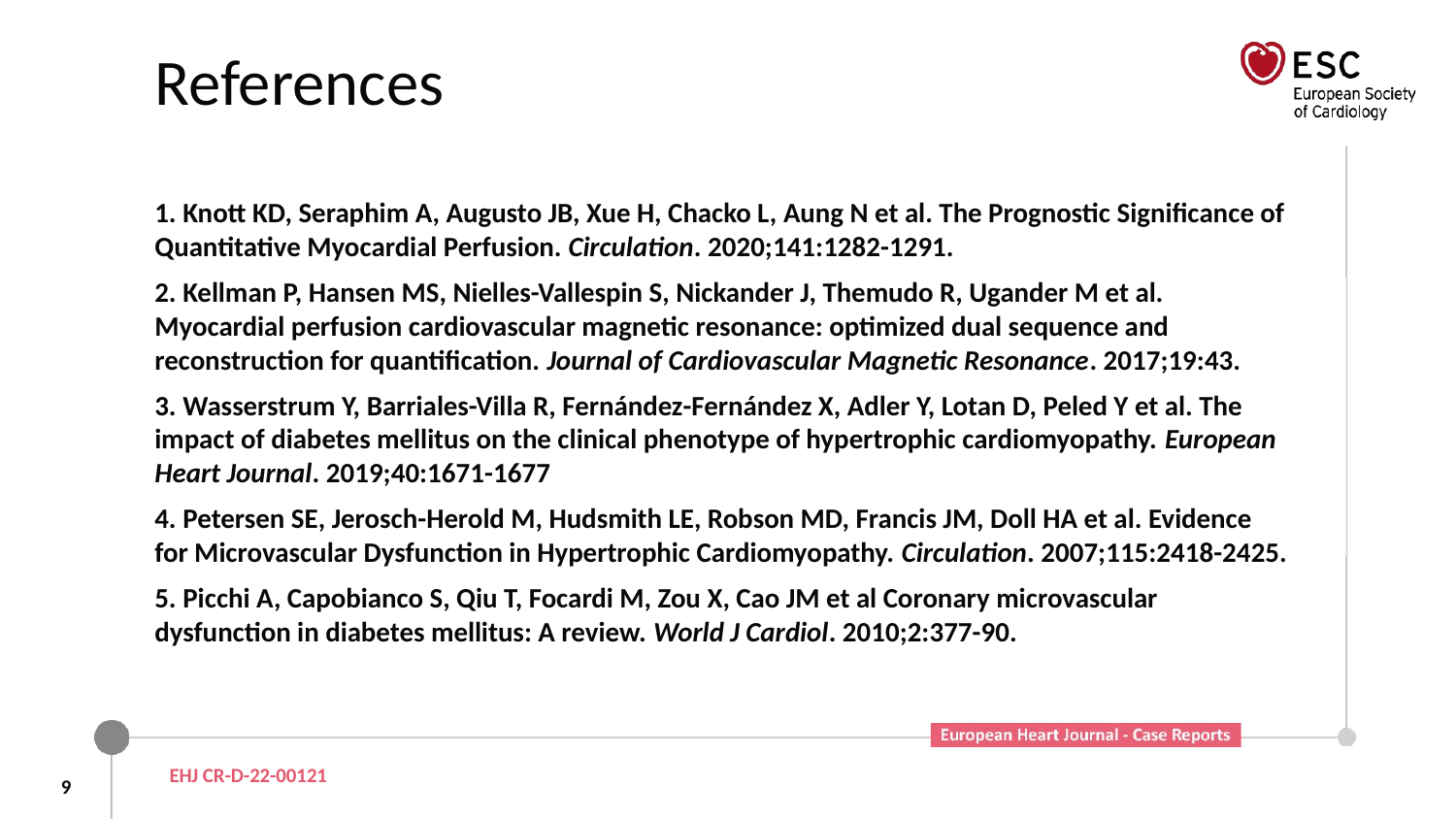

# References
1. Knott KD, Seraphim A, Augusto JB, Xue H, Chacko L, Aung N et al. The Prognostic Significance of Quantitative Myocardial Perfusion. Circulation. 2020;141:1282-1291.
2. Kellman P, Hansen MS, Nielles-Vallespin S, Nickander J, Themudo R, Ugander M et al. Myocardial perfusion cardiovascular magnetic resonance: optimized dual sequence and reconstruction for quantification. Journal of Cardiovascular Magnetic Resonance. 2017;19:43.
3. Wasserstrum Y, Barriales-Villa R, Fernández-Fernández X, Adler Y, Lotan D, Peled Y et al. The impact of diabetes mellitus on the clinical phenotype of hypertrophic cardiomyopathy. European Heart Journal. 2019;40:1671-1677
4. Petersen SE, Jerosch-Herold M, Hudsmith LE, Robson MD, Francis JM, Doll HA et al. Evidence for Microvascular Dysfunction in Hypertrophic Cardiomyopathy. Circulation. 2007;115:2418-2425.
5. Picchi A, Capobianco S, Qiu T, Focardi M, Zou X, Cao JM et al Coronary microvascular dysfunction in diabetes mellitus: A review. World J Cardiol. 2010;2:377-90.
9
EHJ CR-D-22-00121
