# Supplementary figures and images for: A case report of refractory angina in a patient with diabetes and apical hypertrophic cardiomyopathy
Source: Eur Heart J Case Rep. 2022 Aug 16;6(8):ytac347. doi: 10.1093/ehjcr/ytac347 (PMC9425848; doi:10.1093/ehjcr/ytac347)

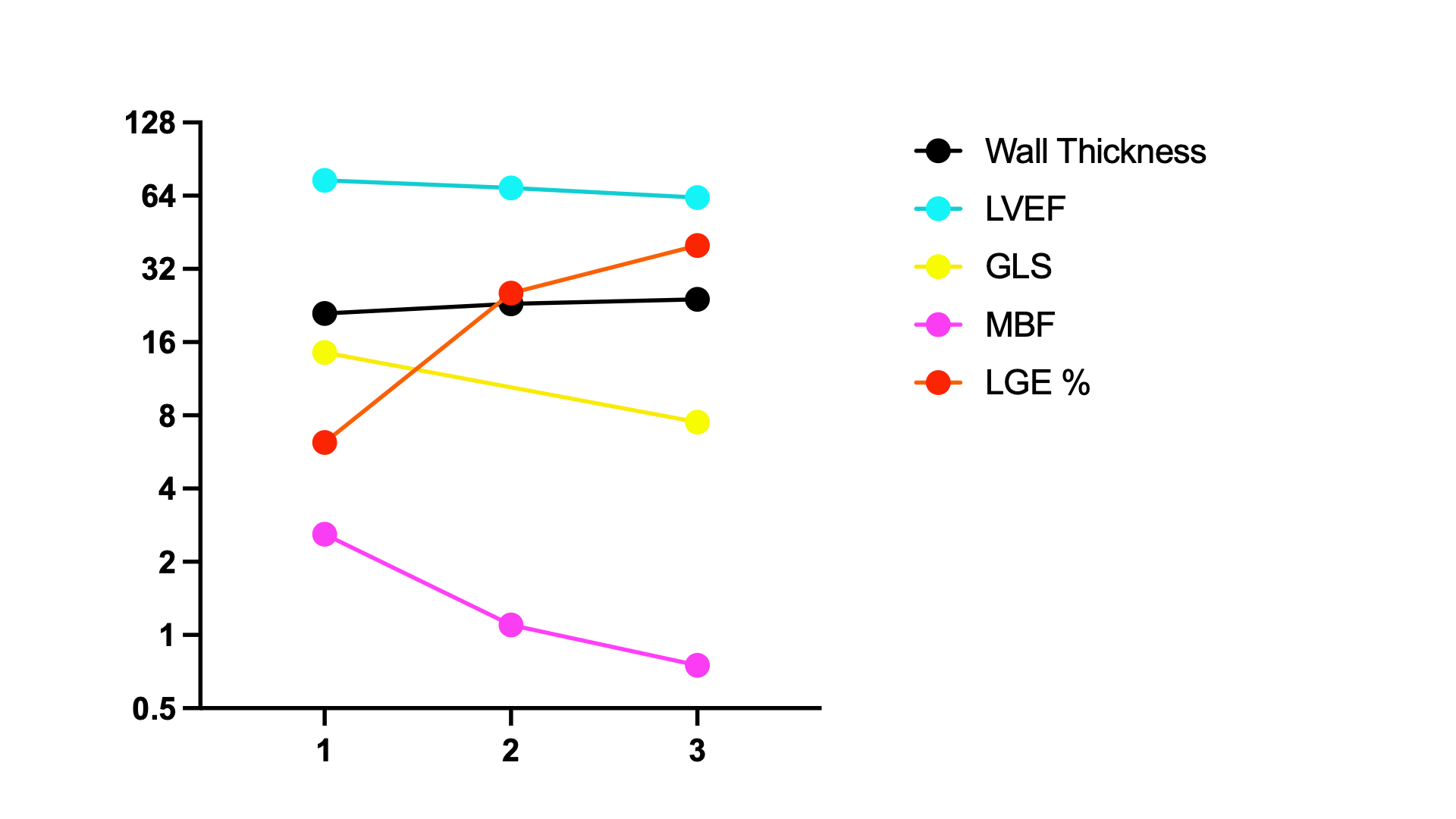

Supplement: ytac347_Supplementary_Data [file ytac347_supplementary_data.zip › EHJ_CaseReport_Figure_5.tiff]
